# Blockchain Revolutionizing in Emergency Medicine: A Scoping Review of Patient Journey through the ED

**DOI:** 10.3390/healthcare11182497

**Published:** 2023-09-08

**Authors:** Tzu-Chi Wu, Chien-Ta Bruce Ho

**Affiliations:** 1 Institute of Technology Management, National Chung-Hsing University, Taichung 40227, Taiwan; bruceho@nchu.edu.tw; 2 Department of Emergency Medicine, Show Chwan Memorial Hospital, Changhua 500009, Taiwan

**Keywords:** blockchain, emergency medicine, IoT, AI, telemedicine

## Abstract

Background: Blockchain technology has revolutionized the healthcare sector, including emergency medicine, by integrating AI, machine learning, and big data, thereby transforming traditional healthcare practices. The increasing utilization and accumulation of personal health data also raises concerns about security and privacy, particularly within emergency medical settings. Method: Our review focused on articles published in databases such as Web of Science, PubMed, and Medline, discussing the revolutionary impact of blockchain technology within the context of the patient journey through the ED. Results: A total of 33 publications met our inclusion criteria. The findings emphasize that blockchain technology primarily finds its applications in data sharing and documentation. The pre-hospital and post-discharge applications stand out as distinctive features compared to other disciplines. Among various platforms, Ethereum and Hyperledger Fabric emerge as the most frequently utilized options, while Proof of Work (PoW) and Proof of Authority (PoA) stand out as the most commonly employed consensus algorithms in this emergency care domain. The ED journey map and two scenarios are presented, exemplifying the most distinctive applications of emergency medicine, and illustrating the potential of blockchain. Challenges such as interoperability, scalability, security, access control, and cost could potentially arise in emergency medical contexts, depending on the specific scenarios. Conclusion: Our study examines the ongoing research on blockchain technology, highlighting its current influence and potential future advancements in optimizing emergency medical services. This approach empowers frontline medical professionals to validate their practices and recognize the transformative potential of blockchain in emergency medical care, ultimately benefiting both patients and healthcare providers.

## 1. Introduction

In recent years, the field of emergency medicine care has witnessed a significant transformation in our conventional healthcare models due to the widespread adoption and application of digital technologies, including artificial intelligence [1], machine learning [2], big data [3], and the metaverse. With the increasing adoption of digital technologies and the growth of healthcare services, there has been a significant increase in the types, velocity, and volume of personal health data, leading to a greater demand for data exchange within the healthcare ecosystem. While big healthcare data holds significant potential, striking a balance between permissible data applications and maintaining security and patients’ rights to privacy presents a formidable challenge [4].

Data security and data ownership have become focal points in the healthcare system [5]. The issue of sensitive healthcare data breaches has become a recurring concern, as evidenced by incidents like the largest healthcare data breaches of 2018, which resulted in the exposure of 13 million total healthcare records and led to significant repercussions [6]. At the same time, sensitive information such as medical history, social security numbers, and financial details faces potential risks. Additionally, with the emergence of personalized healthcare and wearable devices, the ownership of individual healthcare data and the implementation of access control mechanisms have gained paramount significance [5,7]. During this period, it becomes crucial to implement trustworthy technologies while maintaining trust and safety among ecosystem participants. As a result, healthcare institutions are in urgent need of reviewing the current clinical applications and proposing novel and improved solutions to maintain trust and enhance data security within the emergency healthcare ecosystem.

So far, there have been numerous reviews focusing on the applications of blockchain in healthcare. However, across different specialized medical disciplines, unique healthcare sectors and practice settings each possess their own distinct characteristics. Alongside widely utilized electronic medical record applications, the domain of emergency medical care presents specific requirements, encompassing pre-hospital emergency services, inter-hospital transfers, as well as prehospital care for trauma and critical illnesses. These particular aspects have not received a thorough examination in previous literature reviews and have often been overlooked in the majority of evaluations concerning the implementation of blockchain technology in the healthcare sector. Therefore, considering the increasing adoption of blockchain technology, a better understanding of its application and current status in emergency care systems is urgently needed. The study is structured as follows: Section 2 provides an introduction to the background knowledge of blockchain; Section 3 elaborates on the research methods used; Section 4 presents the obtained results; Section 5 discusses the findings; Section 6 addresses future challenges and limitations; and finally, Section 7 concludes the study.

## 2. Background

### 2.1. Blockchain

Blockchain technology, a groundbreaking innovation, has swiftly gained traction, with transformative potential across diverse sectors such as finance, healthcare, information systems, government services, and supply chain management [8,9]. At its core, a blockchain is a decentralized and distributed ledger that enables secure and transparent record-keeping. The emergence of Bitcoin as a cryptocurrency marked the inception of blockchain 1.0 technology [10]. Blockchain 2.0 involves distributed ledgers with smart contracts, whereas Blockchain 3.0 denotes the application of blockchain technology beyond financial contexts, aiming to extend the trustless and decentralized characteristics to other systems, particularly in the healthcare domain [11]. In the following section, we will review the structure and architecture of blockchains, as well as the strengths and weaknesses of various consensus algorithms and platforms in the context of healthcare.

### 2.2. The Structure of a Blockchain

**Figure 1:**
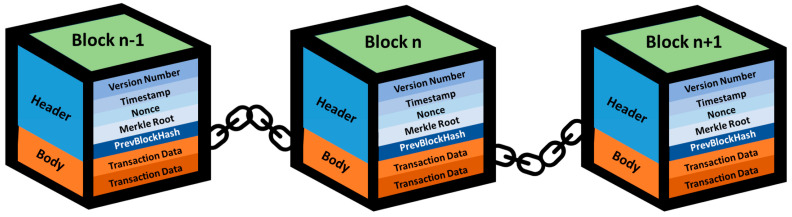
Blockchain architecture.

The blockchain is composed of numerous blocks, which are linked together one by one to create a blockchain. Each block contains two primary components of data: the block header and the block body (Figure 1). The block header functions as metadata for each block within the blockchain, holding critical information about the block. It comprises elements such as the version number, timestamp, nonce, Merkle root, and previous block hash. The version number indicates the protocol version of the block, which is mainly used for protocol upgrades. The timestamp denotes the block’s creation time, while the nonce is a numerical value utilized in proof of work to ensure the block’s hash meets a specified difficulty target. The Merkle root represents a tree-like structure that allows us to obtain the hash value of a sequence of data. Lastly, the previous block hash points to the unique identifier of the preceding block. This connection guarantees a sequential order of blocks and validates the accuracy of previous blocks.

The other component is the block body, which encompasses all the data contributing to the block’s generation. In the context of the Bitcoin blockchain, these data include transaction records. The block body holds the actual information stored within the block and can be considered the block’s payload. A blockchain is established by linking blocks, with each block containing vital metadata in its block header and the actual data in its block body. The block header ensures the integrity and order of blocks within the blockchain, while the block body stores specific data relevant to the blockchain’s purpose, such as transaction records in the case of the Bitcoin blockchain [7,8,12].

### 2.3. Layered Architecture of Blockchain

**Figure 2:**
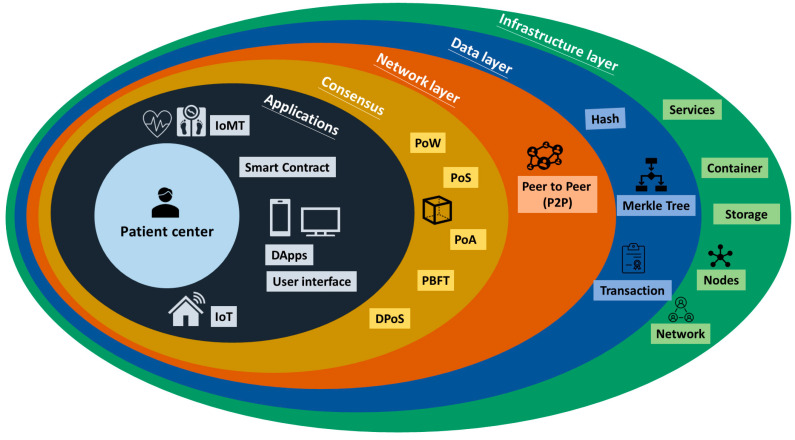
Layered architecture of the blockchain.

In the context of blockchain technology, the architecture typically consists of several key layers that collaborate to enable the functionality of the blockchain network. These layers encompass the infrastructure layer, data layer, network layer, consensus mechanism, and application layer (Figure 2).

The infrastructure layer lays the groundwork by providing the essential elements that underpin the operation of the blockchain network. This encompasses components like the foundational hardware, network connectivity, and data storage. The data layer shoulders the responsibility of storing and managing the factual data recorded on the blockchain. Meanwhile, the network layer serves as the conduit for communication among nodes within the blockchain network. This layer facilitates the dissemination of transactions and blocks across the network. The consensus mechanism, on the other hand, establishes the regulations and protocols that guide the nodes in reaching an accord on the blockchain’s state. It ensures a unanimous consensus among nodes regarding the validity of transactions and the sequence in which they are appended to the blockchain. There are several proposed and implemented consensus protocols, with the three most commonly used ones are Proof of Work (PoW), Proof of Stake (PoS), and Proof of Authority (PoA) [13,14].

Among the consensus algorithms, Proof of Work (PoW) is the most well-known and closely associated with block-chain technology, particularly due to its integration in Bitcoin. Its strengths in healthcare include a well-established consensus algorithm that is decentralized and highly secure, as it does not rely on a central authority to validate transactions. However, it is highly energy-intensive and can be slow, requiring a significant amount of time to validate transactions. Healthcare providers might need to bear higher resource, cost, and time burdens, which could be a drawback, especially in acute care settings. We have summarized the pros and cons of other types of consensus algorithms in healthcare in Table 1.

Lastly, the application layer becomes the realm where developers construct and deploy decentralized applications (DApps) atop the blockchain. This layer interacts with the underlying strata to provide specific functionalities to end-users. These DApps encompass a variety of utilities, such as smart contracts, token issuance, and supply chain tracking, among others [1,15,16,17]. The use of smart contracts in the application layer ensures data provenance and eliminates the need for intermediaries [12]. Smart contracts are self-executing agreements where predefined provisions are encoded in the source code. As these contracts are automatically enforced based on predetermined conditions, they operate without the involvement of third parties or intermediaries. This not only eliminates the need for intermediaries but also provides all stakeholders with a secure and immutable transaction history [8,18].

**Table 1:** Strengths/weaknesses of consensus algorithms.

Consensus Algorithm	Strength	Weakness	Ref.
Proof of Work (PoW)	1. Well-established 2. Highly secure 3. Decentralized	1. Highly energy-intensive 2. Slow, requires time 3. Be vulnerable to centralization	[1,7,8,13,19]
Proof of Stake (PoS)	1. Less energy-intensive 2. Require less computational power 3. Faster (than PoW)	1. Less secure 2. Be vulnerable to centralization 3. Not tested in production environments	
Delegated Proof of Stake (DPoS)	1. Faster (than PoW, PoS) 2. More scalable 3. More decentralized (than PoW, PoS)	1. Less secure 2. Requires active voter 3. Limited scalability	
Proof of Authority (PoA)	1. Faster processing 2. Lower energy consumption 3. Ideal for private blockchains	1. Less decentralized, centralized	
Practical Byzantine Fault Tolerance (PBFT)	1. High speed and efficiency 2. Suitable for permissioned blockchain 3. Tolerates a certain number of faulty nodes	1. Less secure 2. Vulnerable to attacks 3. Limited scalability	

### 2.4. Types of Blockchains

**Figure 3:**
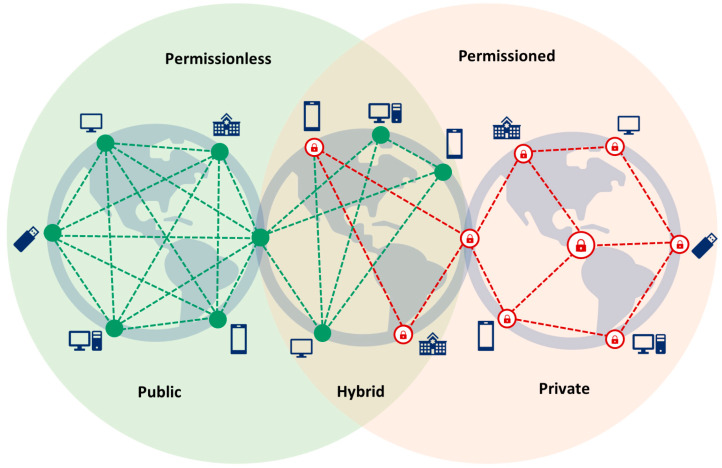
Types of blockchains.

There are three types of blockchains: public, hybrid, and private (Figure 3). Public blockchains, also known as permissionless blockchains, are exemplified by cryptocurrencies like Bitcoin [10] and Ethereum [20], which are extensively employed. Hybrid blockchains, alternatively referred to as consortium blockchains or federated blockchains, exhibit partial centralization. On the other hand, private blockchains, recognized as permissioned blockchains, uphold a distributed network that is frequently centralized [8].

Public blockchains are open to anyone with internet access, allowing anyone to participate in the network. However, they can be less private, slower, and less scalable, particularly in healthcare applications. In contrast, private blockchains restrict participation to known individuals or entities, ensuring controlled and limited access to the blockchain network. Nevertheless, they may be more susceptible to attacks and failures due to their lesser decentralization compared to other blockchain types. Additionally, private blockchains could be burdensome for healthcare entities, as they tend to be more expensive to set up. Hybrid blockchains offer the advantages of both public and private blockchains, yet they are concurrently more intricate and more susceptible to attacks [7,8,21].

### 2.5. Blockchain Platform

There are numerous blockchain platform options available in the current landscape, including Ethereum, Hyperledger, MedRec [22], and MultiChain [23], all of which have been integrated into healthcare systems. Each platform possesses its own strengths and weaknesses. Public blockchain platforms like Ethereum and private ones like Hyperledger are well-known examples. Hyperledger encompasses various established projects, including Hyperledger Fabric, Hyperledger Besu, and Hyperledger Indy, among others [24]. Hyperledger Fabric, in particular, stands out as an enterprise-grade, authorized, secure, and high-performance blockchain network. Functioning as a permissioned blockchain platform, Hyperledger Fabric enables the establishment of private and secure networks tailored for healthcare entities. Its noteworthy scalability makes it a viable option for healthcare applications that involve a substantial volume of transactions. However, it displays less decentralization compared to some other blockchain platforms, which could potentially expose it to increased vulnerability to attacks and operational limitations [24,25].

Conversely, Ethereum, a prominent public blockchain platform, is firmly established with a substantial developer community and a wealth of tools and resources. Nevertheless, it can experience slowness and restricted scalability, which can pose challenges when dealing with a significant number of transactions [7,24,25]. The process of selecting an appropriate off-the-shelf blockchain platform for a specific healthcare or clinical application can be intricate. In previous reviews, a portion of healthcare applications leaned towards the utilization of private or consortium (publicly permissioned) blockchains. This trend is understandable within the healthcare context due to the desire to retain control over access and data recording on the blockchain. Allowing unrestricted writing (adding blocks to the blockchain) or access is not conducive [12,26]. The decision between adopting public or private blockchain networks within the healthcare sector may hinge on specific requirements, regulatory considerations, and the levels of trust established among stakeholders. We have summarized the pros and cons of platforms in healthcare in Table 2.

### 2.6. The Features of Blockchain

**Figure 4:**
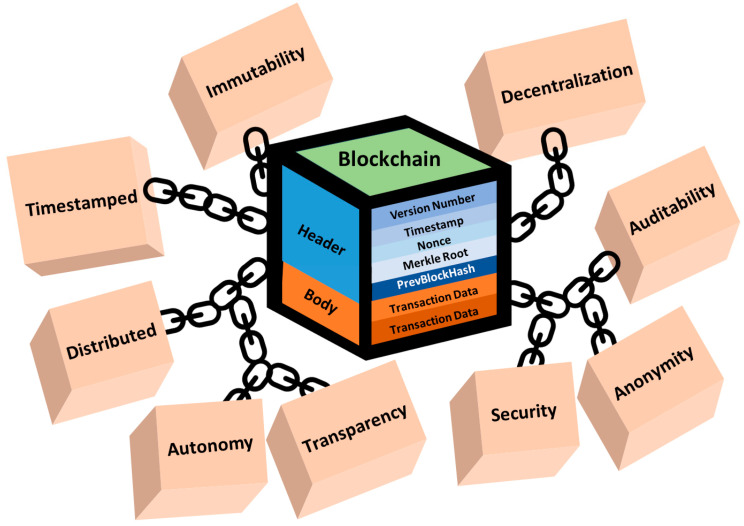
The key features of blockchain technology.

The important characteristics of blockchain, including auditability, anonymity, transparency, immutability, decentralization, and autonomy, have the potential to play a crucial role in ensuring that patients’ personal health information is collected, shared, and utilized appropriately in the context of medical care (Figure 4) [4]. Blockchain’s auditability ensures a tamper-proof ledger of all transactions, accessible for auditing and verification by any network participant. Anonymity offers a heightened level of privacy, allowing users to transact without disclosing their real-world identities, a particularly crucial aspect in sensitive fields like healthcare. The transparency feature furnishes an open and clear record of all transactions, accessible to all network participants. The immutability of the blockchain guarantees an unalterable record of transactions, resistant to any changes or deletions once recorded. Furthermore, its decentralized nature eliminates the reliance on a central authority for transaction validation. Additionally, blockchain operates autonomously, requiring minimal human intervention [7,28].

### 2.7. State-of-the-Art Application and Reviews of Blockchain for Healthcare

Blockchain technology emerges as a groundbreaking force, possessing substantial untapped potential capable of catalyzing significant advancements across diverse healthcare domains, thereby introducing unprecedented transformations. Notably, recent strides in Internet of Things (IoT) technology have facilitated enhanced connectivity, enabling seamless access to critical patient information, vital hospital resources, and wearable devices. The integration of blockchain-driven healthcare further amplifies these advancements. The expansion of health data collection, particularly in the context of remote patient monitoring, encompasses a wide spectrum of health-related information, thereby presenting intricate challenges pertaining to data sharing and accessibility that extend beyond the confines of healthcare facilities. By affording patients complete sovereignty over their historical health records, blockchain assumes a pivotal role in substantially shaping healthcare efficiency and optimizing cost-effectiveness. The empowerment of patients with comprehensive insights into their health records culminates in a remarkable reduction of superfluous documentation and medically redundant tests [5,29,30,31].

Drawing upon the insights gleaned from Table 3, the scholarly community has exhibited a profound interest in probing the influence of blockchain technology within the healthcare landscape. Numerous researchers have undertaken extensive investigations, elucidating the multifaceted impact of blockchain, with a distinct emphasis on its adoption and application across healthcare domains [32]. Furthermore, an array of reviews has predominantly centered on scrutinizing electronic medical records (EMRs) and electronic health records (EHRs) [32,33,34]. Notably, recent trends have showcased an emergent inclination toward reviews that encompass the realms of IoT, AI, and telemedicine applications. However, comprehensive evaluations specifically focusing on blockchain remain relatively scarce, with specialized reviews primarily concentrating on sectors such as dentistry [35], oncology [36], orthopedics [37], radiology [38], and neuroscience [39]. Significantly, the exploration of adoption studies within the realm of emergency care remains conspicuously underrepresented. Each specialized medical discipline introduces unique care paradigms, underscoring the paramount importance and exigency of conducting meticulous and tailored literature reviews tailored to each respective subdomain.

### 2.8. Research Questions

To ensure comprehensive coverage of relevant literature on the topic of interest, we formulated the following initial research questions:

1. What is the level of adoption of blockchain in the field of emergency healthcare, and what are the current applications?
2. From the perspective of the emergency department journey, what are the trends in the application of blockchain in emergency healthcare?
3. What are the specific elements of blockchain technology utilized in publications related to emergency medicine?

From the perspective of the ED journey, we explore how blockchain can be integrated into tracking the workflow of a patient from the pre-hospital stage to triage, treatment planning, and post-discharge care.

## 3. Methodology

### 3.1. Research Protocol

This scoping review aimed to gain insights into the current development and application of blockchain technology in emergency medicine. The review adheres to the Preferred Reporting for Systematic Reviews and Meta-Analysis Extension for Scoping Reviews (PRISMA-ScR) guidelines and is reported accordingly with the PRISMA-ScR checklist [55].

### 3.2. Eligibility Criteria

To be eligible for inclusion in this review, papers needed to focus on the application of blockchain technology in the field of acute medicine. The data sources encompassed the Web of Science (WoS), PubMed, and Medline. The search strings were formulated in accordance with the research domain and the specified research questions. The search terms were as follows:

(Blockchain OR “block chain”) AND (Emergency medicine OR acute medicine OR acute care OR ED)

To make the literature search more comprehensive, we also included other relevant keywords related to the emergency department journey, such as pre-hospital, triage, inter-hospital transfers, and disposition.

(Blockchain OR “block chain”) AND (pre-hospital)
(Blockchain OR “block chain”) AND (triage)
(Blockchain OR “block chain”) AND (inter-hospital transfers OR hospital transfers)
(Blockchain OR “block chain”) AND (disposition OR discharge disposition OR post discharge)

The selection of search terms for this scoping review was based on those commonly used in previous reviews. The term “blockchain” was employed as a consistent search term across all reviews. Additionally, another search term was chosen by the majority authors: “healthcare or medicine OR Medical Management [8,12,26]”. Our choice aimed to narrow the focus of healthcare to the context of emergency medical care within this scoping review. To ensure a comprehensive scope of search, several intermediate stages of the emergency care journey were also incorporated, seeking a more holistic understanding.

Regarding database selection, Web of Science (WOS) was chosen as a recognized authoritative source for social sciences and technology-related information. PubMed and Medline were included due to their coverage of all medical-related literature, establishing them as standard data sources for research papers in health informatics. However, despite the inclusion of these key databases, the comprehensiveness of the sources might have been limited. This limitation will be acknowledged and discussed in the dedicated section. We included studies from the years 2008 to 2023. The reason for choosing the year 2008 as the beginning of the range is the introduction of Bitcoin in 2008, which was the first published application of blockchain technology. The literature search was last updated on 30 June 2023. All reference results were exported into EndNote, and duplicates were subsequently removed.

### 3.3. Selection of Studies

Inclusion criteria:

1. Original research studies.
2. Publications related to blockchain technology in the acute medical care sector.
3. Publications related to blockchain technology are used in the journey of emergency medicine.

Exclusion criteria:

1. Papers without full-text availability.
2. Papers with a focus other than the use of blockchain in the acute medical care sector.
3. Duplicate papers.
4. Editorials, prefaces, paper summaries, summaries of tutorials, correspondences, discussions, comments, readers’ letters, workshops, panels, and poster sessions in the search results.

Two researchers conducted the evaluation collaboratively. The selection procedure encompassed the assessment of titles, abstracts, and complete articles by the first reviewer to ascertain the inclusion of pertinent papers. In cases where the initial reviewer had uncertainties about certain articles, they were subjected to a second evaluation by a separate reviewer. Both reviewers meticulously examined the retrieved articles and documented the data using a pre-established matrix that they jointly formulated.

## 4. Results

**Figure 5:**
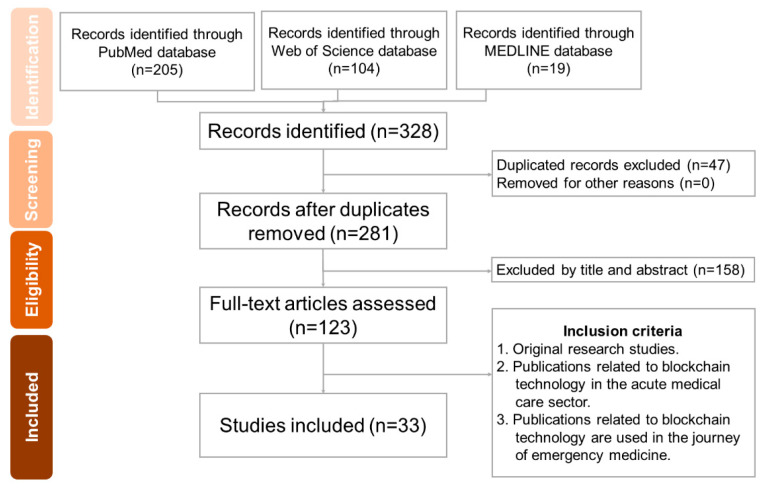
PRISMA-ScR flow diagram.

A total of 328 articles were identified, out of which 47 were duplicates. After applying the inclusion and exclusion criteria, 281 articles were reviewed, and 33 articles remained for further analysis (Figure 5).

The details of 33 articles are provided in Table 4. The findings reveal that data sharing and documentation, including personal health records (PHR) and electronic health records, are the primary areas where blockchain technology is applied. Notably, the pre-hospital application stands out as a distinctive aspect compared to other disciplines, encompassing hospital referrals, trauma scene alarm systems, secure drone information dissemination, and even the dispatch of emergency materials during disasters. Additionally, AI and deep learning are the most commonly paired technologies with blockchain, while smart phones, IoT, and IoMT have generated numerous emerging data sources.

Among the various platforms, Ethereum (n = 10) and Hyperledger Fabric (n = 7) emerge as the most frequently utilized ones in this domain. Regarding the type of blockchain, several papers failed to define their approach, especially in the journey of medical care and post-disposition, but it appears that public blockchains and consortiums are the preferred types, especially in pre-hospital domains. As for the consensus algorithm, the most frequently used consensus algorithm in the included publications was PoW, accounting for 27% (9/33) of the published works, followed by PoA, which accounted for 15% (5/33).

Regarding smart contracts, 66.7% (22/33) of the included studies utilized them for various functionalities, while the remaining studies did not specify if smart contracts were employed or not. Privacy, security, access control, and interoperability are all important issues addressed in these publications. Interoperability, data integrity consistency, and cost could be challenges in the future for the emergency medicine journey.

## 5. Discussion

In our review, we have observed a growing number of blockchain applications in emergency medical care. The most prevalent application involves medical record management, followed by healthcare delivery, post-discharge monitoring, and pre-hospital care. The former has rapidly developed due to its universal demand across the medical field, and there is a gradual increase in the adoption of blockchain in specialized emergency care applications. With the advancement of IoT and 5G technologies, post-discharge monitoring and pre-hospital care hold the potential to significantly benefit from blockchain technology.

Regarding the utilization of consensus algorithms, each consensus method possesses its own strengths and weaknesses, dependent on specific characteristics [13]. Among these methods, Proof of Work (PoW) and Proof of Authority (PoA) stand out as the most commonly employed consensus algorithms in our review. The notable strength of PoW lies in its security, which is a critical and prioritized factor in healthcare applications. However, PoW’s energy-intensive and slow nature might pose challenges in healthcare scenarios that demand swift and efficient data processing. Similarly, PoS algorithms are well-established but offer relatively lower security. Consequently, they may not align well with healthcare applications that demand both security and speedy transaction processing in the future. PoA, on the other hand, presents opportunities through its attributes of speed, lower energy consumption, and privacy. It streamlines the consensus process, enhances energy efficiency, and restricts participation to predefined authorities or validators, making it suitable for healthcare settings [19].

Similar to the findings of previous reviews, Ethereum and Hyperledger Fabric are the primary platforms of focus. Ethereum’s strength lies in its well-established nature, flexibility, and user friendliness, which facilitate smooth applications in acute medical care. However, in the future, challenges may arise due to its potential to be slower, more energy-intensive, and less scalable compared to other blockchain platforms. As the volume of real-time individual data increases in pre-hospital care and post-discharge IoT scenarios, healthcare will face greater challenges. On the other hand, Hyperledger Fabric boasts high scalability, making it particularly suitable for healthcare applications with a significant transaction load. Its standout feature is its emphasis on privacy and security, allowing organizations to maintain control over data and transaction access, which proves to be a significant advantage in healthcare applications. Nonetheless, Hyperledger Fabric’s complexity and higher learning curve present challenges and signify areas where efforts can be directed for future emergency medical education and promotion.

**Figure 6:**
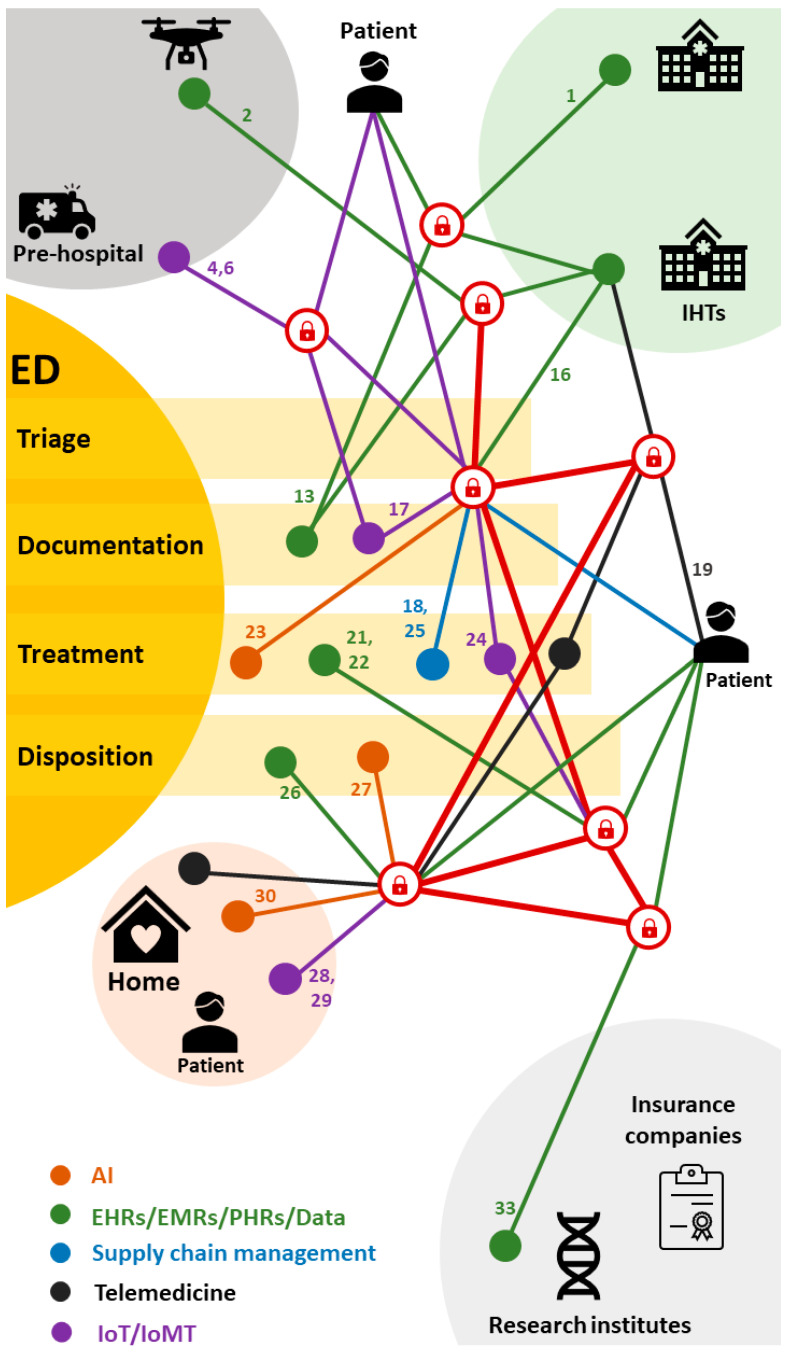
Emergency department journey map (ID Number of the included studies in Table 4).

Next, we have created an emergency department journey map (Figure 6) and allocated the “ID Numbers” of selected studies to their respective stages within the journey. The emergency department (ED) map illustrates a patient’s journey, commencing with pre-arrival incidents like car accidents or heart attacks, advancing to pre-hospital care or transfers, followed by the arrival at the emergency entrance for triage, subsequent assessment and treatment, disposition, and ultimately returning home. The inclusion of ID numbers along this pathway signifies the placement and relevance of blockchain applications along the emergency department journey. This aids in providing a clearer comprehension of the current status and potential growth of blockchain applications at different phases of the journey. In the forthcoming discussion, we will delve into the current scenario and future prospects of blockchain applications based on the emergency department journey map. Finally, we will provide examples focused on two distinctive scenarios in emergency medical care: pre-hospital and disposition at home.

### 5.1. Pre-Hospital

In the application of blockchain, pre-hospital emergency medical care stands out as the most distinctive aspect compared to other specialties. It comprises two major components: emergency medical services (EMS) and inter-hospital transfers (IHTs), with the former being particularly noteworthy. EMS serves a paramount role in preserving human lives and reducing mortality and morbidity rates [89]. Various studies have demonstrated that accurate and early detection by EMS plays a crucial role in timely hospital management and admission for emergencies such as ST-elevation myocardial infarction, acute stroke, and trauma [90]. In the context of EMS, it involves the collection of pre-hospital individual data and assessment results, which may be transmitted to data centers or cloud systems through the internet. These data could be accessed by hospitals or individuals with different needs. Additionally, with the increasing popularity of electronic transmission technologies and social platforms, some social media platforms have been utilized as channels for data transmission, effectively reducing treatment time [90].

Consequently, there is a growing body of research utilizing artificial intelligence (AI) algorithms based on deep learning to enhance the quality of pre-hospital emergency care. Many studies have confirmed that AI in emergency medicine improves accuracy and efficiency, and reduces time-to-treatment for the detection of out-of-hospital cardiac arrests (OHCA), stroke detection [91], and EKG interpretation for ST-elevation myocardial infarction (STEMI) [92]. Unfortunately, most of the data obtained from the aforementioned scenarios may not have been adequately protected.

In these processes, various forms of data such as patients’ basic information, medical records, clinical images, audio recordings, and video footage are often collected, stored, and accessed for further data analysis. The security of structured, semi-structured, and unstructured data obtained from patients, including considerations related to storage, access, management personnel, application usage, and data disposal, is often underestimated. Furthermore, the utilization of social media as a transmission platform raises concerns about data ownership, the rights of social media platforms to access and use personal data, as well as the monitoring of individuals who have access to such data. Additionally, the application of artificial intelligence involves direct data collection from individuals, leading to uncertainties and ambiguity regarding the scope of data collection and sharing with third parties, which may not be clear to patients.

At this juncture, blockchain technology can unleash its powerful capabilities, encompassing secure storage and protection of patients’ personal health data to prevent unauthorized access or tampering. Additionally, it facilitates improved data sharing and access control mechanisms, limited to authorized personnel within the EMS system or the intended receiving hospital. This ensures sensitive data can only be accessed by those with authorization. Furthermore, blockchain can be employed to track and verify the origin and authenticity of data collected outside the hospital, guaranteeing the accuracy and credibility of such data. This, in turn, aids healthcare providers in making precise medical decisions based on the trustworthy information gathered beyond hospital premises.

**Figure 7:**
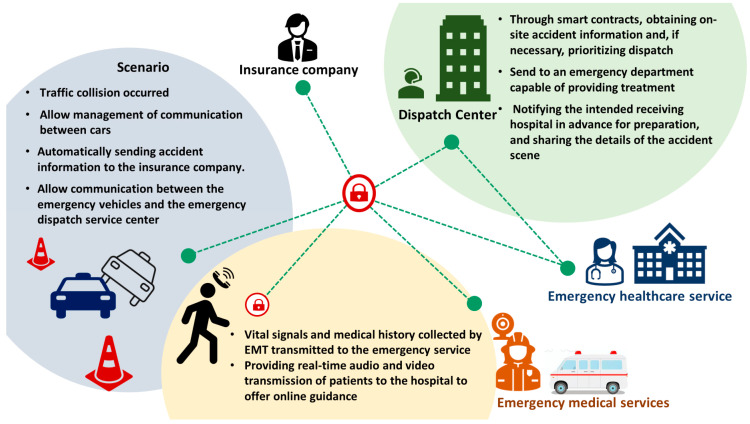
Examples of blockchain usage scenarios in pre-hospital care.

So far, from the moment a car accident occurs, blockchain technology has been put to use. Amel Ksibi et al. propose a system that combines the Internet of Vehicles (IoV) and the Internet of Medical Things (IoMT) concepts with blockchain technology, enabling emergency vehicles to arrive at the accident scene promptly and speeding up interventions in emergency cases. Additionally, this system ensures the security of patient data [61]. Moreover, blockchain technology has already been applied in conjunction with fifth-generation wireless networks in the context of newborn emergency transport [93]. Fang et al. leverage blockchain technology to ensure data security and immutability, while exploring how blockchain can enable trusted data sharing and collaboration among multiple stakeholders to enhance the efficiency and accuracy of emergency transport [93]. We have partially combined the instances mentioned earlier and are now providing examples of blockchain utilization in pre-hospital care (Figure 7).

Inter-hospital transfers (IHTs) play a crucial role in healthcare systems by enhancing access and efficiency in care delivery [94]. However, in many countries, such as England, patients often receive treatment from multiple hospital trusts that utilize different record systems, resulting in significant barriers to inter-hospital data sharing and interoperability [95]. Furthermore, the exchange of personal health data among hospitals faces technological and individual obstacles, including resistance to information sharing, privacy concerns, and divergent interests [74,96]. Some hospital trusts still rely on paper records, in-house-developed electronic health record (EHR) systems, or disparate electronic systems. The lack of secure and transparent personal health data poses concerns, considering that effective IHTs are integral to improving access, reducing costs, and allocating resources appropriately within healthcare systems [94].

Systematic review studies indicate that research on blockchain technology in healthcare has increased, and one of the most commonly used applications is for data sharing, which aligns with our findings [26]. To address these challenges, an example solution is proposed by Hasavari and Song, who propose a solution based on blockchain technology, utilizing a secure file transfer protocol (FTPS) for secure file transfer, coupled with blockchain as the ultimate data source [7]. In this approach, emergency-relevant medical data of patients are securely uploaded to a TLS server integrated with the Fabric blockchain, using advanced tools for scheduling and automating the uploading process. Once stored on the ledger, the data are replicated and distributed to other authorized network members, such as hospitals or healthcare personnel, based on predefined policies, ensuring a consistent and comprehensive view of the patient’s health data [7]. Numerous similar frameworks have been proposed, and by leveraging blockchain technology, improvements can be made in facilitating coordination among electronic health record systems, developing targeted approaches to enhance interoperability, and improving the effectiveness and efficiency of data sharing between hospitals [95]. In the realm of pre-hospital care, there exists a scarcity of pertinent reviews at present. We contend that velocity, cost, and real-time responsiveness will emerge as the foremost challenges in the future, while compatibility stands as a primary concern for potential inter-hospital transfers.

### 5.2. ED Triage

The triage process is crucial to effectively managing modern emergency departments [97]. The information gathered during triage assists medical personnel in assessing the condition of patients and determining appropriate treatment. However, in our review, there was no specific focus on blockchain research in triage. Nevertheless, there has been a growing trend in the application of various digital technologies in this area. For example, various digital technologies have been applied, including digitally-automated pre-hospital triage in the USA [98], a computerized triage system known as the automated triage system in Malaysia, decision-making support systems (DMSS) for triage, triage chatbots, and the application of AI in triage [99,100,101,102], which are used to aid assessment and improve the accuracy and efficiency of triage. Many personal data, including audio, some images, and even videos, are transmitted, stored, and analyzed. However, security and privacy concerns arise when collecting and processing such data, particularly regarding the confidentiality of patients’ personal health information.

By storing clinical images/videos or their hashes on a blockchain, they can be easily shared among different healthcare systems and providers. Integrating pre-hospital data with ED triage, the blockchain serves as a mechanism to authenticate requesting parties, such as emergency department physicians, specialist physicians, or dispatch centers, ensuring their authorization to access specific data based on a permissioned list. In the future, the utilization of blockchain technology has the potential to facilitate secure storage and sharing of vast amounts of data during triage, resulting in enhanced security and reliability of the information [103].

### 5.3. Documentation and Data Exchange

Accurate and complete medical data, including electronic medical records (EMRs) and electronic health records (EHRs), are valuable assets for patients and healthcare providers. With the development of electronic technology in recent years, there have been advancements not only in storage formats and security but also in recording methods. The method of medical record recording has also evolved, transcending traditional computer typing or handwriting, with the integration of natural language processing artificial intelligence systems capable of automatically drafting a health record based on a recording of a patient-emergency physician interaction as it occurs [104]. In the past, healthcare settings and providers have been hesitant to share medical data due to security concerns. Additionally, conventional healthcare systems often employ inadequate security measures or systems, resulting in potential risks to overall system security, especially with the introduction of some digital technologies, such as AI, which significantly increase the risks.

Blockchain presents a promising opportunity in this context, as data sharing is highly secure and verifiable through cryptographic principles [12]. Currently, similar to past research, the most prevalent applications of blockchain technology in healthcare primarily revolve around electronic medical records [105]. Many review articles emphasize that electronic health records (EHR) and personal health records (PHR) are the primary areas targeted for blockchain implementation [8]. These serve as a digital record of a patient’s medical history, and blockchain technology efficiently addresses the limitations of traditional systems by offering reliable, accurate, and secure data storage and exchanges [106].

In general, a patient-centric node on a permissioned blockchain, which operates based on business logic, requires robust identity management, accountability, access control, and authorization [7]. Healthcare providers need to obtain enrollment and transaction certificates to connect to and access resources on the Fabric network. By leveraging blockchain and smart contracts, the system empowers patients to efficiently enforce permission-based access control policies for their data and enables the sharing of previous health records with emergency doctors in critical situations. The execution steps involve a doctor or hospital initiating an emergency request to the medical center’s infrastructure, which then processes the data at the edge services. Subsequently, the data are formatted and identified for transactional purposes. Additionally, when an emergency request is initiated, the system sends the request information to the transaction manager for storage and enters a waiting state. Finally, the request is processed with an approval status, granting access to the patient’s database to the doctor and other healthcare providers [7,107]. When emergency physicians evaluate patients and record their cases, blockchain technology can be applied to store all patient medical histories in a centralized location, facilitating faster and more transparent access for authorized parties and reducing manual errors [108]. In this manner, we can effectively integrate personal medical information that is dispersed across multiple hospitals, enabling patients to access and utilize their health records freely in both their daily lives and during emergencies [67].

Based on our review, it is evident that interoperability, scalability, and access control constitute the principal challenges in the forthcoming landscape of electronic health records (EHR) and personal health records (PHR). This observation aligns with the outcomes of the majority of analogous systemic reviews conducted in the past [32,33]. Allowing patients to have certain control and autonomy over their data is crucial, enabling them to decide which nodes to grant access to. Additionally, the transparency of data in the blockchain ensures reliable and accessible information, instilling confidence in patients during inter-hospital transfers or when seeking a second opinion from another doctor [105,109]. A secure and interoperable health system built on blockchain technology has the potential to facilitate intelligent and optimized exchange of medical data among diverse entities, such as hospitals of different levels, thereby reducing redundant medical procedures and resource wastage [66].

### 5.4. Treatment

#### 5.4.1. Drugs and Medical Devices

After the evaluation by the emergency physician, the physician will issue medical orders for treatment, which may include the use of medications and procedures. In terms of blockchain and supply chain management, medication safety is a primary application area. Counterfeit drugs have presented a significant challenge to the global pharmaceutical industry, with pharmaceutical companies facing difficulties in tracking their products throughout the supply chain process [110]. The pharmaceutical supply chain has become increasingly complex due to its involvement in life-saving treatments and the participation of various stakeholders, including pharmaceutical manufacturers, dealers, distributors, patients, information service providers, and regulatory agencies [18,111]. In this context, blockchain technology emerges as a solution. Blockchain can trace and track the drug delivery at every phase, from the supplier’s raw materials to manufacturing, distribution, hospitals, and patients. For instance, Modum.io is a startup that utilizes IoT sensor devices and blockchain technology to ensure the immutability of data and public accessibility of temperature records, while also reducing operational costs in the pharmaceutical supply chain [112]. In the context of medicinal drug supply chain management, blockchain provides a crucial and secure platform to mitigate vulnerabilities, address fraudulent attacks, enhance data transparency, and improve product traceability [80,105].

Many medical devices also face similar challenges, but relevant applications in emergency medical care are currently less common. The lack of collaboration among enterprises in the medical device logistics service supply chain and the absence of unified data management norms and standards for data collection among logistics enterprises have resulted in fragmented supply chain processes [113]. As a result, medical devices or supplies face significant challenges regarding storage and transportation conditions, logistics environment, timeliness, end-to-end tracking and monitoring, as well as the management capabilities of logistics service providers [113,114]. Yu and Fu proposed a comprehensive medical equipment management information system that integrates blockchain technology with the full life cycle theory [115]. Such applications not only ensure quality control for medical devices or supplies but also integrate with existing hospital nodes and patient nodes.

In the clinical treatment of patients, blockchain technology has been applied in other specialties for monitoring and tracking certain implanted medical devices. We believe there is an opportunity to securely record and track vital information related to implanted medical devices, such as central venous catheters (CVC), endotracheal tubes, or Foley catheters, at the emergency department in the future [103]. This includes details such as the device model, placement date, and longevity, ensuring accurate and reliable documentation throughout the patient’s healthcare journey. Furthermore, this application of blockchain technology facilitates seamless continuity of care as patients transition between different healthcare settings. To sum up, the system has the opportunity to track the usage of specific medications or devices by individual patients, providing valuable insights into the production and transportation processes of raw materials and enhancing the overall quality of care for emergency patients.

#### 5.4.2. Consent

During the treatment process, consent forms are often utilized. However, in our review, we didn’t find any relevant literature on this topic. This could be attributed to the search terms used or the fact that current research is more conceptual in nature rather than specifically focusing on emergency care. Nevertheless, the implementation of blockchain in consent forms still holds great potential, including its application in informed consent documents, do-not-resuscitate (DNR) directives, and the shared decision-making process between healthcare providers and patients [116]. Blockchain-based informed consent, combined with smart contracts, simplifies the process of obtaining informed consent, enhances identity management, and improves data quality. This technology enables the digitization of paper consent forms and facilitates the signing of digital medical consent forms [117]. The general procedure involves patients registering their authentic identities and receiving validation from a third-party verifier. They can then utilize blockchain certificates and private keys to complete and sign the consent forms. The signed content is securely stored within the blockchain network, ensuring the integrity of the signing records. In contrast to traditional paper consent forms, digital consent forms offer advantages such as immutability and long-term preservation. They also overcome geographical limitations by allowing for remote signing. Furthermore, timestamps are an essential feature of the blockchain. They enable the recording of signing time and sequence among various roles, which is not achievable with paper consent forms [118].

### 5.5. Disposition

After receiving treatment, patients may have various options, such as discharge, admission, or transfer, depending on their medical condition and the risk assessment conducted by the emergency physician. They may also need to proceed with medical expense payments. Our review indicates that there are already some blockchain applications in post-discharge vital signs monitoring and readmission prediction that are utilized to protect patient data security. For instance, the blood sample-based ED return technology is implemented in the ED of a hospital in South Korea, combining big data cloud, machine learning, and blockchain to predict ED return probability [82]. Direct application of payment and insurance in the emergency department is scarce, but there have been some conceptual studies in other fields that have great potential for future applications.

Not only does blockchain have the potential to integrate billing and payment systems, it can also potentially synchronize insurance underwriting with hospital operations. This integration can provide the latest information to patients, healthcare providers, and insurance companies, and it also offers an opportunity to avoid health insurance fraud [119]. It comprehensively scrutinizes each payment transaction while also predicting the insurance amount, thereby alleviating the workload of insurance auditors [30]. Furthermore, through patient-authorized access, insurance companies can directly receive the necessary documents for insurance claims, streamlining the entire claims process. Taking Taiwan as an example, the current process for filing insurance claims after receiving emergency medical care requires patients to first apply for a diagnosis report and relevant documents from the hospital, which are then submitted to the insurance company. After assessment by the insurer, the underwriting process can proceed, which may take one to two months. If the use of blockchain for reimbursement purposes is implemented in the future, it is believed that it can significantly improve patient satisfaction with emergency medical care.

Finally, in terms of the payment system, there are currently no specific applications focusing on post-emergency department payment journeys. However, blockchain-based payment systems have been proposed in other fields. In current healthcare systems, payment settlement between patients and healthcare providers often relies on centralized third-party services. However, these centralized methods are associated with slow processing times, inefficiency, and a lack of transparency [48]. To overcome these limitations, blockchain platforms offer the opportunity for cryptocurrency-based payment systems that can provide a fast, secure, transparent, and auditable payment environment following an emergency department visit.

### 5.6. Home

**Figure 8:**
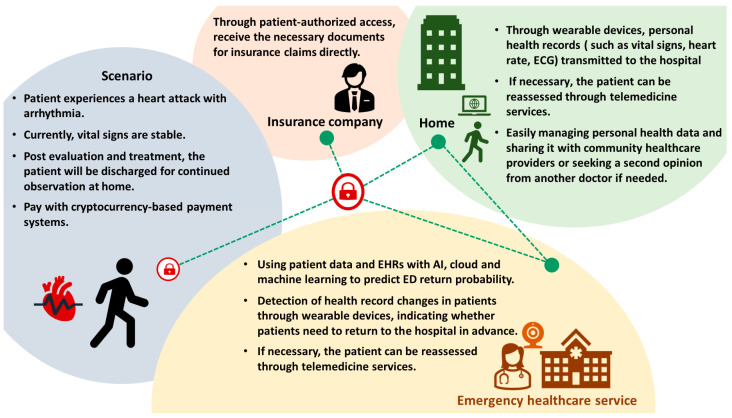
Examples of blockchain usage scenarios in disposition and home.

After the patient’s discharge, care continues at home and transitions back into the community’s healthcare system. The use of wearable devices is becoming increasingly common, including applications in IoT, AIoT, and telemedicine. These technologies are extending the reach of emergency care into the community. Additionally, the use of blockchain technology is also on the rise. In the subsequent sections, we will delve into the applications of IoT, AI, and telemedicine during the final phase of the emergency department journey, which is the patient’s return home. Additionally, we will present illustrative scenarios showcasing the utilization of blockchain technology in this stage of the emergency care journey (Figure 8).

#### 5.6.1. IOT

IoT (Internet of Things) serves as a prominent method of collecting data from various networked resources and interconnected devices. In the realm of healthcare, IoT devices play a crucial role in providing real-time sensory data from patients, which is recognized as an extension of healthcare. It is also the major application of blockchain in the stage of home. They offer efficient components for coordinating care between hospitals and community services in the management of both acute and chronic patients [120]. In acute medical care, IoT holds the potential to extend emergency healthcare by enabling post-discharge monitoring of patients who may require extended observation or are at a higher risk of disease. Through wearable devices, personal health records and vital signs can be transmitted to the hospital, enabling relevant personnel to remotely monitor patients’ health. This not only contributes to reducing overcrowding in the emergency department but also addresses the issue of prolonged stays.

The utilization of wearable devices and personal health data brings substantial and growing value to the emergency healthcare sector. However, it also presents challenges such as single points of failure, mistrust, data manipulation, tampering, and privacy concerns [121]. The combination of blockchain and IoT holds immense potential and can yield significant benefits in distributed applications involving sensitive patient data [122]. These events can be securely sent to patients and healthcare providers, giving patients the individual right to select who can access and view their medical information [123]. However, in our review, there remains a significant disparity among each study. The design of the pre-built or theoretical part of the IoT network necessitates the consideration of numerous parameters. Therefore, a consortium blockchain with additional features will be essential to accommodate IoT requirements. As highlighted by Zubaydi et al., consensus algorithms stand out as a primary limitation or drawback in such models, as the utilization of generalized algorithms hampers the system’s ability to function at its full potential [54].

Ensuring the secure and convenient sharing of personal health data is crucial for enhancing interaction and collaboration within and beyond the healthcare industry [124]. Simić et al. demonstrate how IoT devices can be utilized as data sources for real-time or near-real-time data collection, with blockchain ensuring secure access and data exchange between institutions [122]. Additionally, Liang et al. propose a user-centric medical data exchange solution that leverages a mobile application to gather data from wearable devices. This collected data are then shared with healthcare providers, insurance companies, and research institutes through a permissioned blockchain network [5,117]. By utilizing such an autonomous network with distributed storage, built upon the foundation of blockchain, it extends its reach to connect emergency healthcare with post-discharge care, home healthcare, and personalized precision medicine [30].

#### 5.6.2. AI

The inclusion of artificial intelligence (AI) in the discussion is primarily due to its wide-ranging applications in emergency healthcare in recent years. AI has been extensively utilized throughout the emergency department (ED) journey, encompassing pre-hospital EMS, emergency department triage, diagnosis by emergency physicians, medical record documentation, disease outcome prediction, and even post-discharge monitoring. Big data not only empowers AI but also mandates its utilization for data interpretation, comprehension, and decision-making to maximize favorable outcomes [125,126]. However, many AI implementations depend on centralized datasets and servers, which expose them to the risks of data alteration and loss, consequently leading to potentially unreliable and untrustworthy outcomes [103]. Integrating blockchain databases with AI and IoT not only enhances data security but also improves the performance of machine learning models, achieving a win-win situation in terms of security, usability, and scalability [1].

#### 5.6.3. Telemedicine

Telemedicine is considered one of the most significant innovative responses during the COVID-19 pandemic, and its importance has been gradually increasing in recent years. In the field of emergency medicine, telemedicine extends beyond the assessment of confirmed patients during the pandemic and encompasses pre-hospital emergency services, teleconsulting, as well as post-discharge monitoring and readmission prevention [127]. However, traditional telemedicine systems mostly rely on outdated methods for storage and maintenance [48]. The centralization of existing telemedicine systems presents a significant challenge as it introduces the risk of a single point of failure [128]. Additionally, medico-legal concerns have raised questions regarding the relevance and clarity of communication during the informed consent process, as well as data security issues. These challenges are expected to be addressed through the application of blockchain technology.

In the healthcare sector, the readiness to embrace blockchain technology is still at an early stage. Ahmad et al. demonstrate the practicality of blockchain technology in the telehealth domain, which can bring about significant improvements in terms of reliability, traceability, immutability, and transparency [48]. However, limited research has been conducted specifically to explore the perspectives and adoption trends of emergency medicine professionals regarding blockchain. In general, emergency physicians routinely manage critical patient data in situations where time is of the essence. In such a context, blockchain holds significant promise not only for enhancing decision-making processes but also for safeguarding patient privacy and data security. Nevertheless, there are challenges that must be overcome, not only the ones mentioned in the previous review but also scalability and interoperability issues. These hurdles must be tackled before blockchain can effectively establish itself as a viable tool within our dynamic and rapidly evolving emergency healthcare landscape.

## 6. Challenges and Limitations

The integration of blockchain technology into healthcare offers a viable solution, yet it is still in its early stages and faces specific limitations and challenges. Some of these challenges include cost, complexity, the emerging nature of the technology, the absence of established security and privacy standards, as well as concerns regarding security [48].

To begin with, implementing a blockchain system in emergency care or hospital settings involves significant costs due to the substantial number of transactions that need to be processed in such environments. Speed is also a factor to consider, particularly in emergency medical situations where platforms like Ethereum and consensus algorithms such as Proof of Work (PoW) might encounter limitations in real-time applicability due to their slower processing speeds. Secondly, the complex nature of diverse blockchain implementations that utilize varying underlying technologies can impede seamless collaboration among different systems. Standardization becomes a critical issue that needs attention to ensure smooth data exchange and cooperation across various healthcare systems. This is especially crucial in emergency units that need to interact with pre-hospital, EMS systems, community healthcare groups, and patients, highlighting the challenge of achieving compatibility. It’s only through compatibility that frontline emergency medical personnel can genuinely be assisted rather than hindered. Thirdly, while pursuing efficiency, cost-effectiveness, and scalability, concerns about privacy and confidentiality arise. This also leads to different platforms and algorithms having varying applicability in different scenarios. Public blockchains offer advantages such as high decentralization but are vulnerable to exposing stored information if vulnerabilities are detected in their underlying encryption schemes [127]. Lastly, security is a significant concern, as the loss of a private key could render data permanently unreadable. Moreover, these systems are inherently susceptible to a type of attack known as the 51% attack [129].

Through our review, we have compiled the current status of blockchain applications in emergency medical care and presented blockchain-based scenarios, particularly in various domains within this field. Many of these solutions have been successfully implemented in real-world emergency medical contexts, while others remain promising concepts that are either still hypothetical or in the theoretical stage. However, our study does have certain limitations. Firstly, methodological decisions have introduced constraints in the research process, including the utilization of specific search terms and queries to gather evidence and a focused approach on emergency medicine, which may not encompass all the features of blockchain. Secondly, the potential incompleteness of databases could hinder the representation of the comprehensive landscape of current blockchain applications. Thirdly, the majority of studies are proof-of-concept trials that require additional supporting evidence. Future research could concentrate on applications before hospital admission and after discharge to optimally leverage the value of emergency care medicine. Additionally, investigations into treatment, informed consent, and educational applications remain limited, underscoring the need for greater attention and involvement from professionals in the field of emergency medicine.

## 7. Conclusions

The scoping review examines and discusses significant applications of blockchain technology within the realm of emergency medicine. A notable aspect that sets emergency medicine apart from other fields is the utilization of blockchain in pre-hospital and post-discharge contexts. Consensus algorithms, blockchain platforms, and types of blockchains lack a universal advantage; their effectiveness varies depending on the distinct demands of different emergency care scenarios, each possessing unique strengths, weaknesses, and requirements. Presently, blockchain implementation primarily revolves around electronic health records (EHR) and personal health records (PHR), yet potential challenges loom, including scalability, interconnecting disparate systems, and inadequate system interoperability. The integration of blockchain with the Internet of Things (IoT) is gaining traction, broadening the scope and services of emergency medical care, particularly during the “home” phase of the emergency department journey.

Healthcare providers must deeply comprehend the existing challenges and prerequisites in acute medical care, a vital foundation for productive collaboration with blockchain experts and the successful deployment of blockchain solutions. The shift towards patient-centered, blockchain-enabled care is increasingly apparent. The amalgamation of blockchain technology into emergency medicine holds the promise of enhancing patient-centered interoperability and optimizing overall efficiency and security in healthcare data management. This potential is further exemplified by the streamlined access to patient data and the facilitation of real-time communication among patients, emergency physicians, and other specialists. Furthermore, the inherent attributes of auditability and transparency confer significant advantages in ensuring the secure and trustworthy exchange of medical data among various stakeholders, encompassing patients, healthcare providers, and insurance entities. Looking ahead, focused endeavors should be channeled towards addressing challenges like costs, scalability, security, access control, and standardization, depending on the demands of different scenarios. These endeavors will contribute to the widespread integration of blockchain in acute medical care.

## Figures and Tables

**Table 2:** Strengths/weaknesses of platforms.

Platform	Strength	Weakness	Ref.
Hyperledger	1. Permissioned blockchain platform 2. Supports smart contracts 3. Highly scalable	1. Less decentralized 2. Still being developed, smaller developer community 3. Complex to set up	[1,7,23,24,25,27]
Ethereum	1. Well-established 2. Supports smart contracts 3. Supports dApps 4. Highly decentralized	1. Highly energy-intensive 2. Slow 3. Less scalable 4. High cost	
MedRec	1. Permissioned blockchain 2. Interoperability 3. Secure and decentralized platform for storing and sharing medical records	1. Limited scalability 2. Compatibility 3. Challenges related to regulatory compliance 4. Data standardization	
MultiChain	1. Flexible 2. Customizable platform	1. Smaller developer community 2. High technical expertise to set up and maintain	

**Table 3:** Previous reviews on blockchain in healthcare.

Study	Year	Review Type	Context
[26]	2018	Systematic Review	Analysis of state-of-the-art blockchain research in the field of healthcare.
[27]	2019	Systematic Review	Showcased the ongoing research into the application of blockchain technology in the healthcare sector.
[40]	2019	Comprehensive Review	Conducting a review to analyze and map the research landscape of blockchain technologies, particularly their applications in healthcare.
[41]	2019	Review	Presented the applications and challenges of blockchain technology faced by the healthcare industry.
[42]	2019	Review	Examine the concept of blockchain technology and the challenges associated with its adoption, and a review of the recent implementations.
[24]	2019	Systematic Review	Introduce healthcare and biomedical blockchain applications along with their underlying platforms and compare popular platforms.
[43]	2019	Systematic Review	Focus on blockchain-based electronic medical record systems.
[44]	2020	Systematic Literature Review	Current implications for the use of blockchain technology for improving healthcare processes.
[8]	2020	Scoping Review	Provided a review of the utilization and proposal of blockchain to enhance processes and services within the healthcare sector.
[31]	2020	Systematic Review	Evaluate blockchain technology research for patient care and propose a research agenda.
[32]	2020	Systematic Review	Blockchain technology in electronic health records.
[45]	2020	Scoping Review	Examine and classify the advantages and risks associated with implementing blockchain technology in healthcare systems.
[36]	2020	Systematic Literature Review	Examine the motivations, advantages, limitations, and future challenges of implementing advanced distributed ledger technology in the field of oncology.
[33]	2021	Systematic Review	Blockchain Personal Health Records
[46]	2021	Systematic Review	The potential of blockchain technology in healthcare applications, including those related to COVID-19 and non-COVID-19 contexts.
[37]	2021	Systematic Review	Blockchain technology in orthopedic healthcare.
[47]	2021	Narrative Review	Summarizing current and future uses of blockchain in healthcare and upcoming research directions.
[48]	2021	Review	Illustrate cases showcasing the practical application of blockchain technology in the domain of telehealth and telemedicine and delve into the associated challenges.
[49]	2021	Analytical Review	To comprehend the full range of blockchain implementations and explore the potential of blockchain solutions in healthcare.
[39]	2021	Review	Blockchain technology in medicine and neurology.
[34]	2022	Scoping Review	Highlight the potential and obstacles of incorporating blockchain technology into EHR systems.
[38]	2022	Review	Blockchain technology in radiology research and clinical practice.
[50]	2023	Scoping Review	Explores the benefits, challenges, and patient empowerment gaps in integrating blockchain technology within the existing healthcare landscape, particularly in the patient-centric blockchain-based framework of the EHR paradigm.
[51]	2023	Review	Examine the significance and constraints associated with the utilization of blockchain technologies for enhancing healthcare operations.
[52]	2023	Systematic Review	Concentrate on privacy-enhancing techniques utilizing blockchain and federated learning in telemedicine.
[53]	2023	Systematic Review	Summarizing existing studies on blockchain adoption in healthcare.
[35]	2023	Literature Review	Blockchain technology in dental healthcare.
[54]	2023	Systematic Literature Review	Approaches involving the integration of blockchain and IoT are specifically aimed at addressing certain security and privacy-related issues.

**Table 4:** Overview of the included studies.

Id, (Year), Reference	Journey of ED	Technic or Application	Main Challenge/Limitation	Consensus Algorithm/Smart Contracts	Types of Blockchains	Blockchain Platform
1. (2019) [56]	Pre-hospital (referral)	EMR, EHR (medical referral service)	Scalability	Proof of Authority (PoA) and PoET/Yes	Consortium blockchain	Go Ethereum
2. (2021) [57]	Pre-hospital	UAV	Privacy, scalability, security/cost, and capacity	PoW/Yes	Public blockchain	Ethereum
3. (2022) [58]	Pre-hospital	UAV	Security and effectiveness	Not defined/hashed time locked contract	Not defined	Not defined
4. (2022) [59]	Pre-hospital	IoT	Trust and transparency	Not defined/Yes	Private blockchain	Hyperledger Fabric
5. (2023) [60]	Pre-hospital	Dispatch of emergency materials	Automated, reinforcement learning	PoS, DPos, authorization proof, PBFT/Yes	Public, consortium, and private blockchain	Hyperledger Fabric
6. (2023) [61]	Pre-hospital	Internet of Vehicles (IoV) and IoMT	Data storage, confidentiality, and security	PoW/Yes	Public and consortium	Ethereum
7. (2019) [62]	Documentation, data exchange	Personal Health Record (blood sugar)	Data privacy regulations	PoW/Yes	Hybrid blockchain	Not defined
8. (2020) [63]	Documentation, data exchange	EHR	Privacy, scalability, and availability	Not defined/Yes	Consortium blockchain	Hyperledger Fabric
9. (2020) [64]	Documentation, data exchange	COVID-19 data sharing, ML	Privacy, integrity, and scalability	Not defined/Yes	Public-permissioned blockchain	MedRec
10. (2020) [65]	Documentation, data exchange	EHR	Secure, trustable data sharing	PoW and PBFT/Yes	Private blockchain	Hyperledger Fabric
11. (2021) [66]	Documentation, data exchange	IoMT, edge computing, MEdge-Chain architecture	Efficient, secure connectivity	Proof of Stake (DPoS)/Yes	Permissioned blockchain	Not defined
12. (2021) [67]	Documentation, data exchange	Personal Health Record	Data integration, data ownership, and privacy	Proof of elapsed time consensus algorithm/Yes	Private blockchain	Hyperledger Fabric
13. (2022) [68]	Documentation, data exchange	Personal Health Record (PHR)	Retrieve cross-hospital medical data, low adoption rate	Proof of Authority (PoA)/Yes	Consortium blockchain	iWellChain Framework/Ethereum
14. (2022) [69]	Documentation, data exchange	Patient data sharing and storage	Legal ramifications, delayed transactions, and costs	Proof of Authority (PoA)/Yes	Private blockchain	Ethereum
15. (2022) [70]	Documentation, data exchange	Transfer from EHR to patient PHR	Performance and energy consumption	Proof of Authority (PoA)/Yes	Private blockchain	Ethereum
16. (2023) [71]	Documentation, data exchange	IoMT, deep learning, AI	Data security	Not defined/Yes	Not defined	Not defined
17. (2023) [72]	Documentation, data exchange	IoMT, healthcare smartphone	Security, reliability	PBFT/Yes	Consortium blockchain	Hyperledger Fabric
18. (2022) [73]	Medical care	Supply chain of medicines	Solid, secure, decentralized, transparent	Not defined/Yes	Private/public blockchain	Hyperledger Fabric
19. (2021) [74]	Medical care	Telemedicine	Scalable issue	Proof of work (PoW)/No	Not defined	Not defined
20. (2020) [75]	Medical care	Supply chain for medical supplies	Transparency, improving efficiency	Not defined/No	Not defined	Not defined
21. (2022) [76]	Medical care	Acute craniocerebral trauma anesthesia	Medical data sharing	Not defined/No	Not defined	Not defined
22. (2022) [77]	Medical care	Stroke care information management	Decreasing wait times, secure	Not defined/No	Not defined	Not defined
23. (2022) [78]	Medical care	CNN for COVID image detection	Data security	Proof of Work (PoW)/No	Permissioned blockchain	Not defined
24. (2022) [79]	Medical care (blood pressure sensor)	Fog computing, IoMT	Packet error rate, reliability, and throughput	Proof of Work (PoW)/Yes	Private blockchain	Ethereum
25. (2023) [80]	Medical care	Blockchain with IoT, supply chain management	Quality assurance, tracing, transparency, and security	Proof of Work (PoW)/Yes	Public blockchain	Ethereum
26. (2020) [81]	Disposition	COVID surveillance and case tracking system	Standardization and interoperability	Proof of Authority (PoA)/No	Private blockchain	Ethereum
27. (2021) [82]	Disposition	IoMT, machine learning	Limited information	Not defined/No	Not defined	Not defined
28. (2021) [83]	Home	IoMT, transfer personal data to hospital system	Decreased time, precise, and cost-effective	Proof of Work (PoW)/Yes	Public/Private Blockchain	Ethereum
29. (2021) [84]	Home	IoT, interplanetary file system (IPFS)	Security and privacy	Not defined/Yes	Hybrid blockchain	Not defined
30. (2021) [85]	Home	AI, IoT	Privacy and security	Not defined/No	Not defined	Not defined
31. (2021) [86]	Home	IoT data and AIoT	Cost	Not defined/No	Not defined	Not defined
32. (2018) [87]	Others	Health Professions Education	Trust and transparency	Not defined/No	Not defined	Not defined
33. (2019) [88]	Others	Clinical trials	Policy change	Not defined/No	Not defined	Not defined

## Data Availability

Not applicable.
